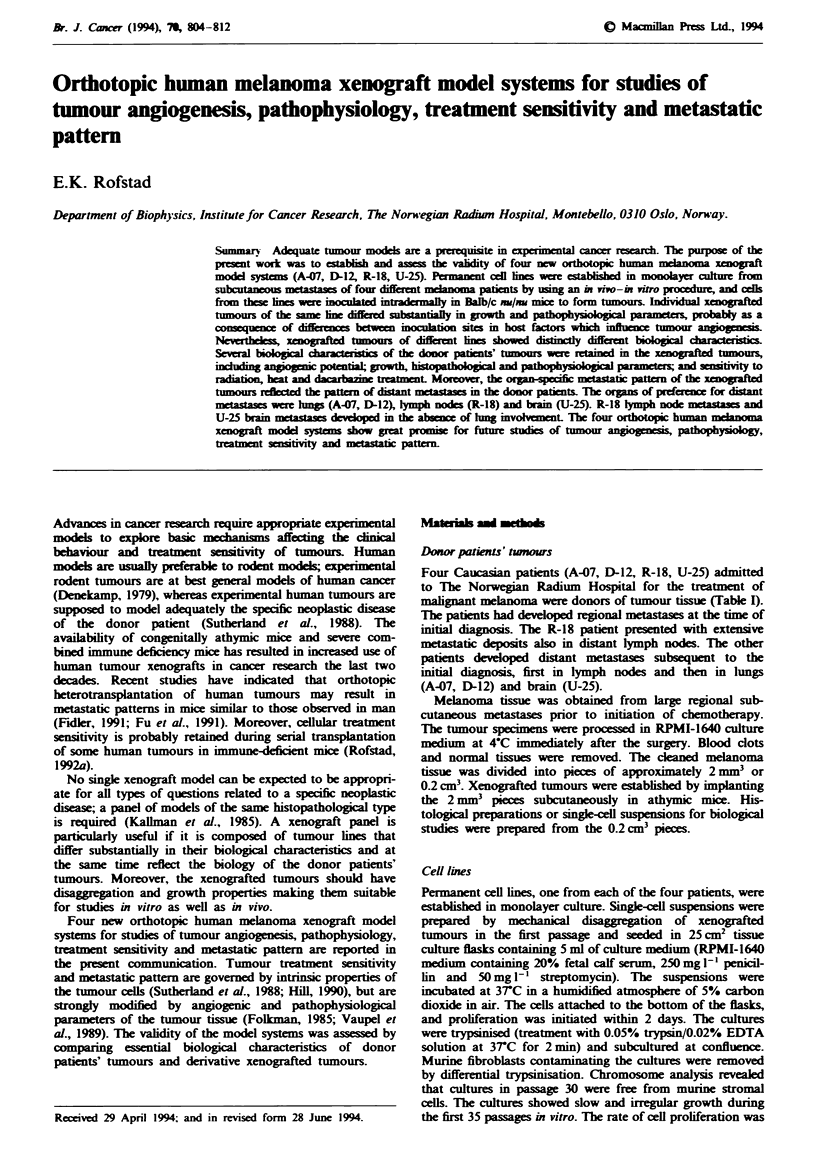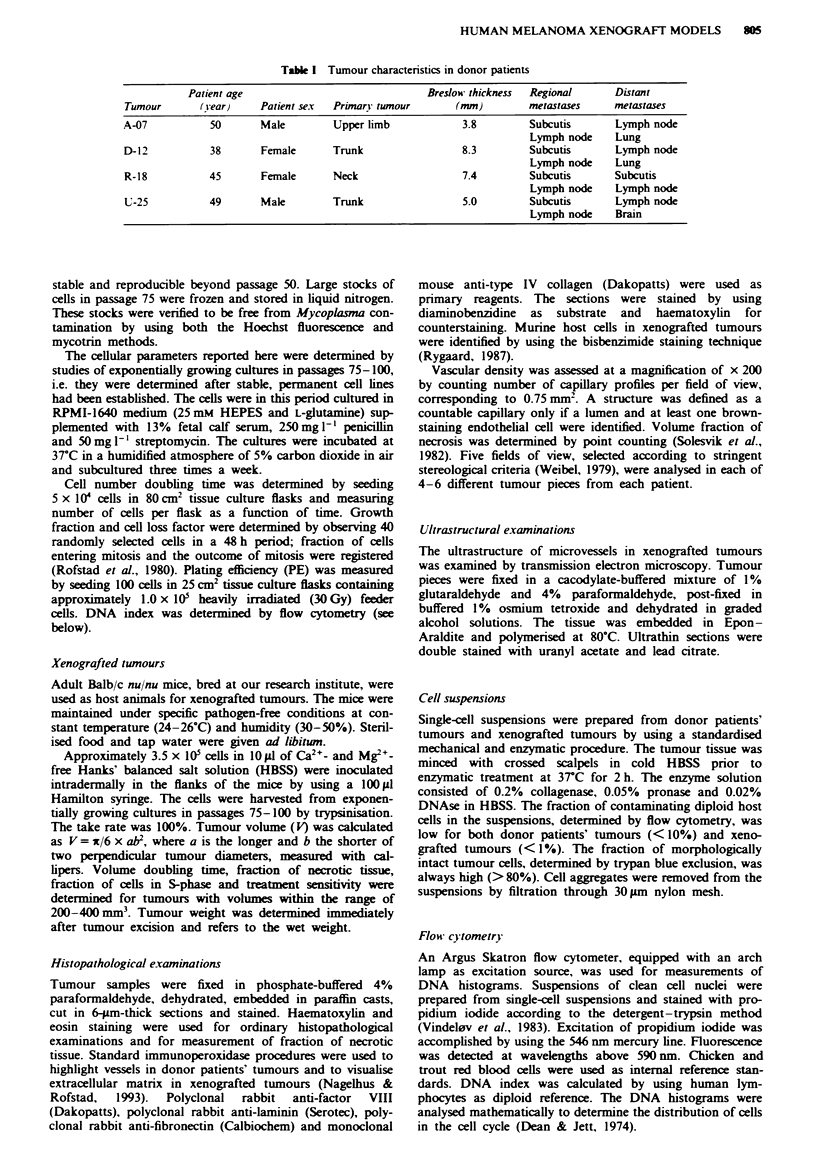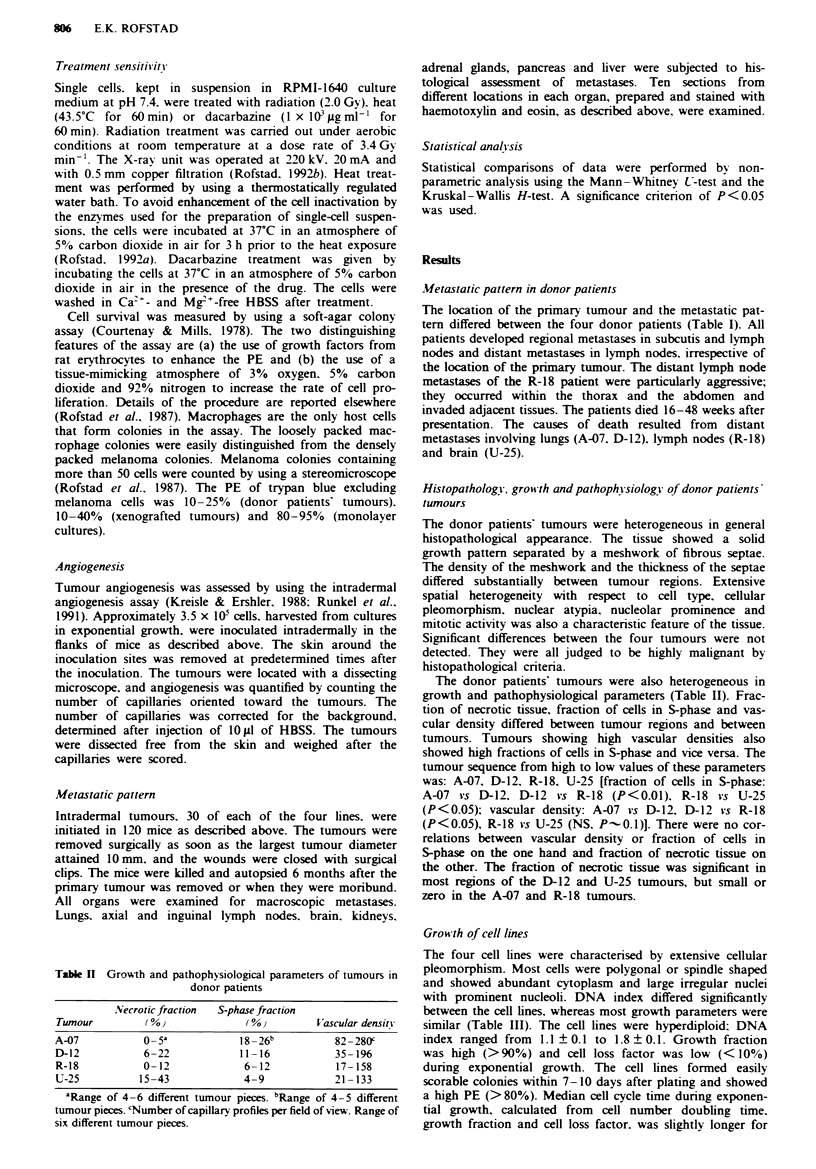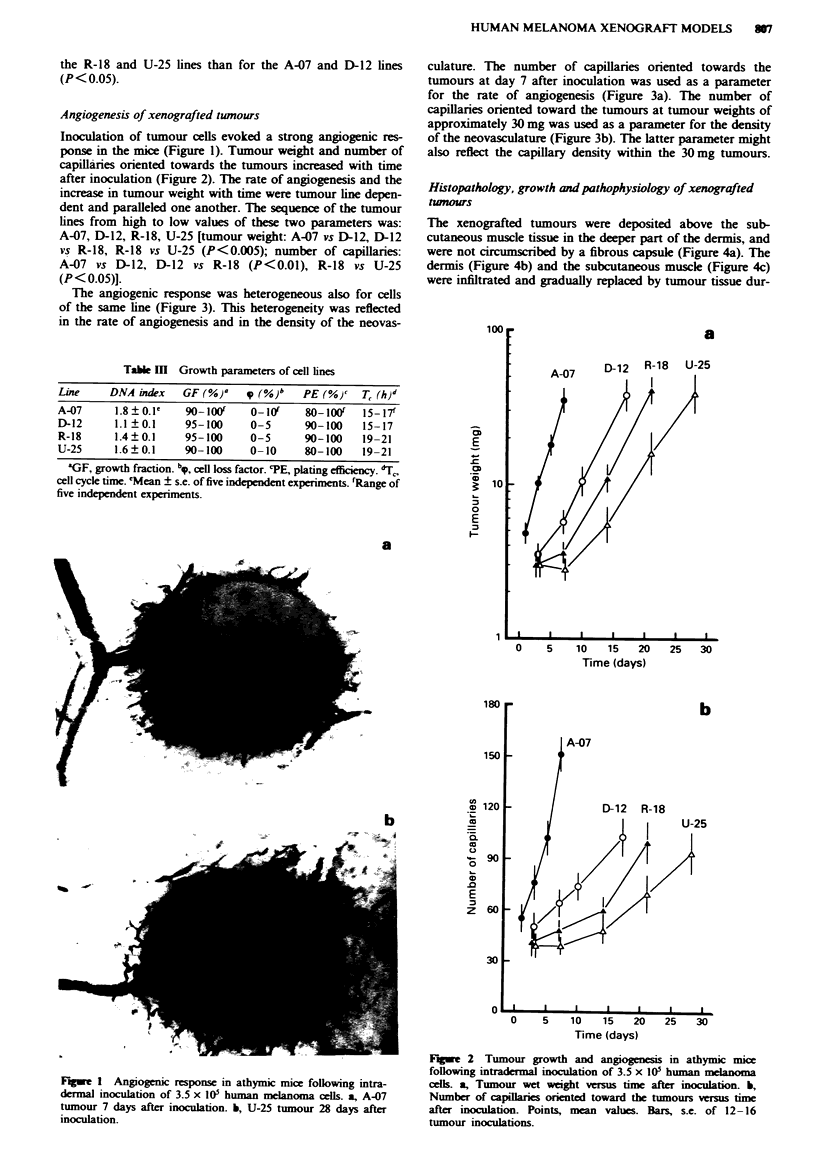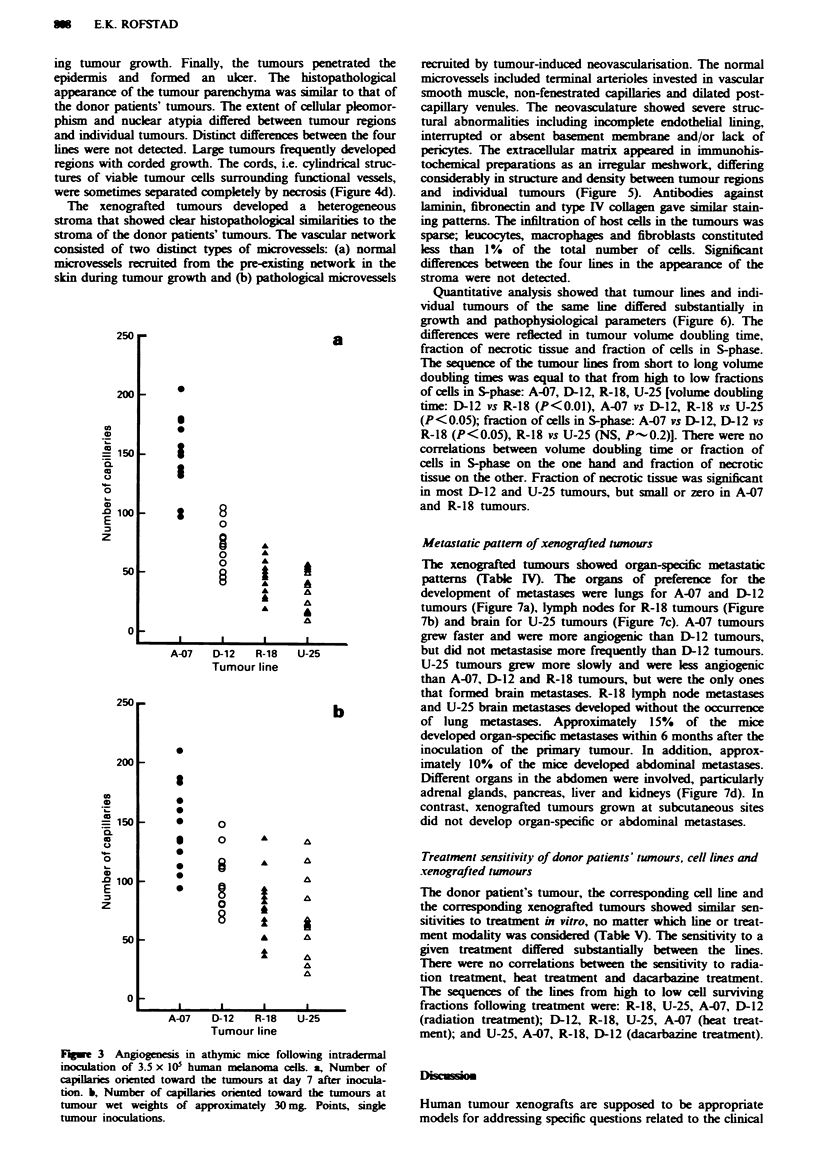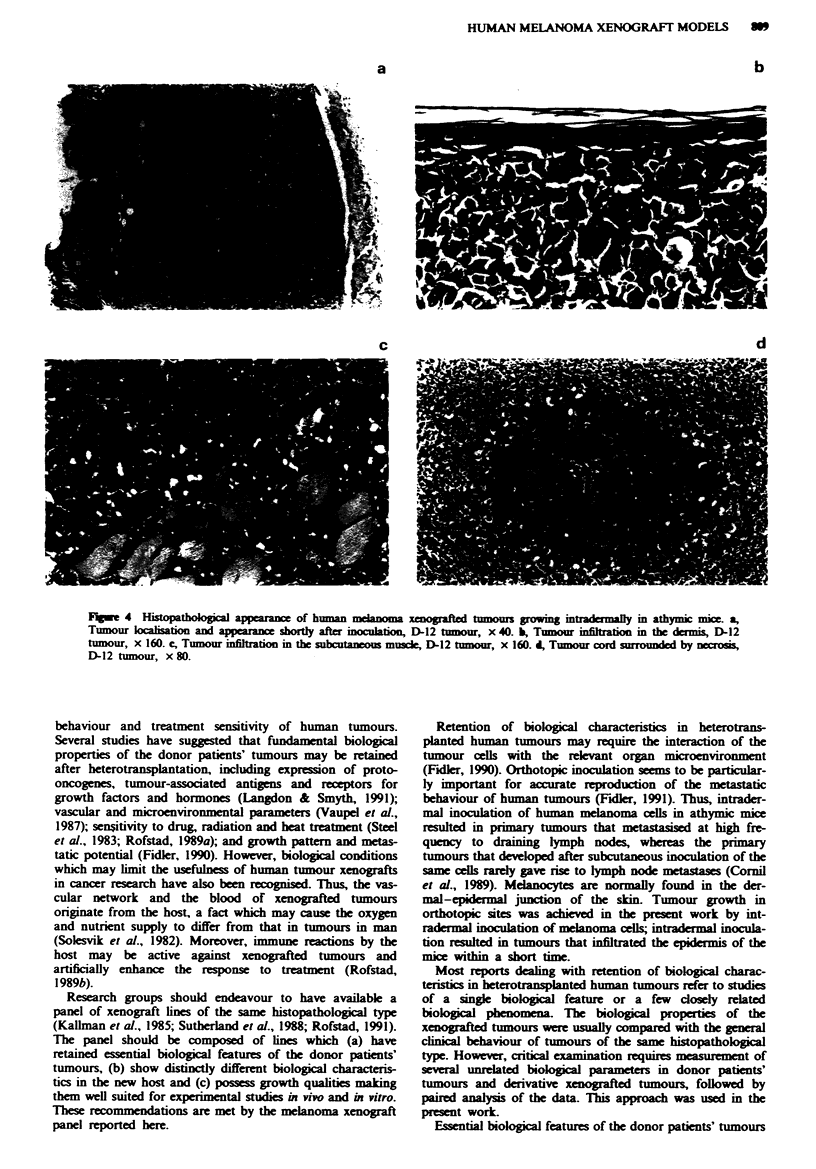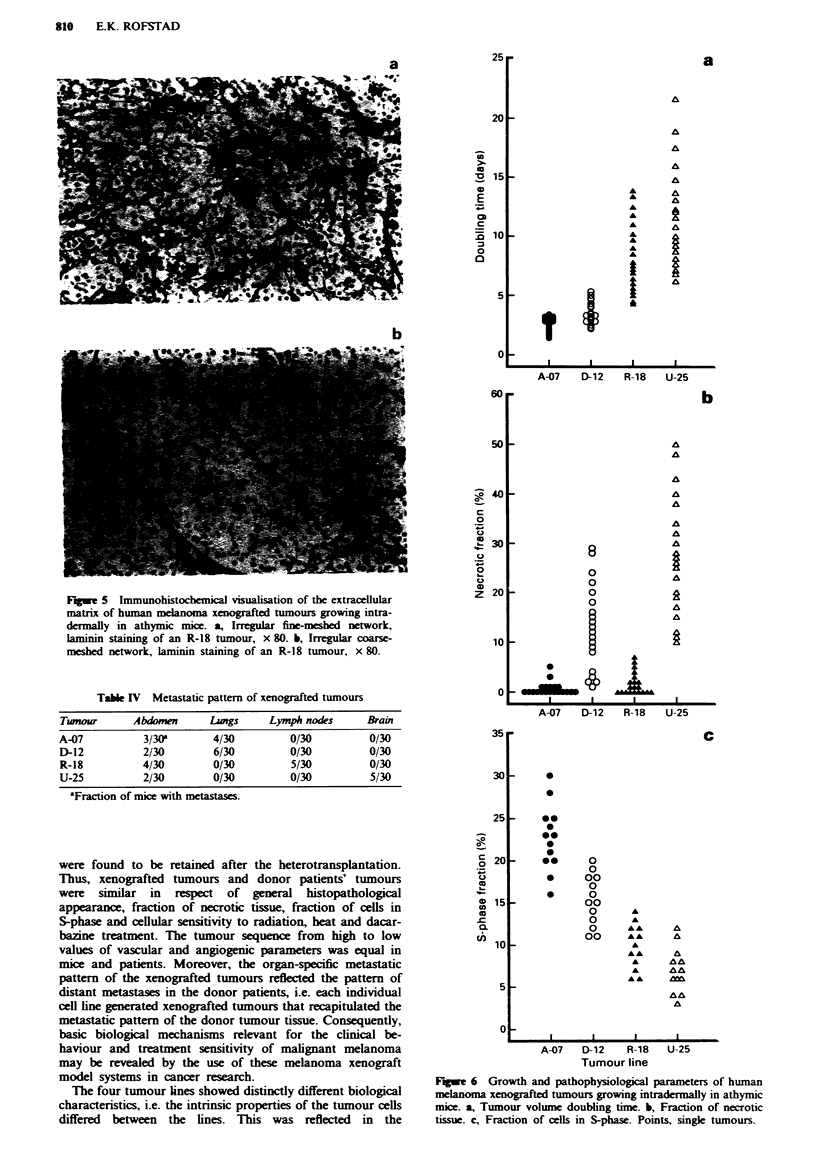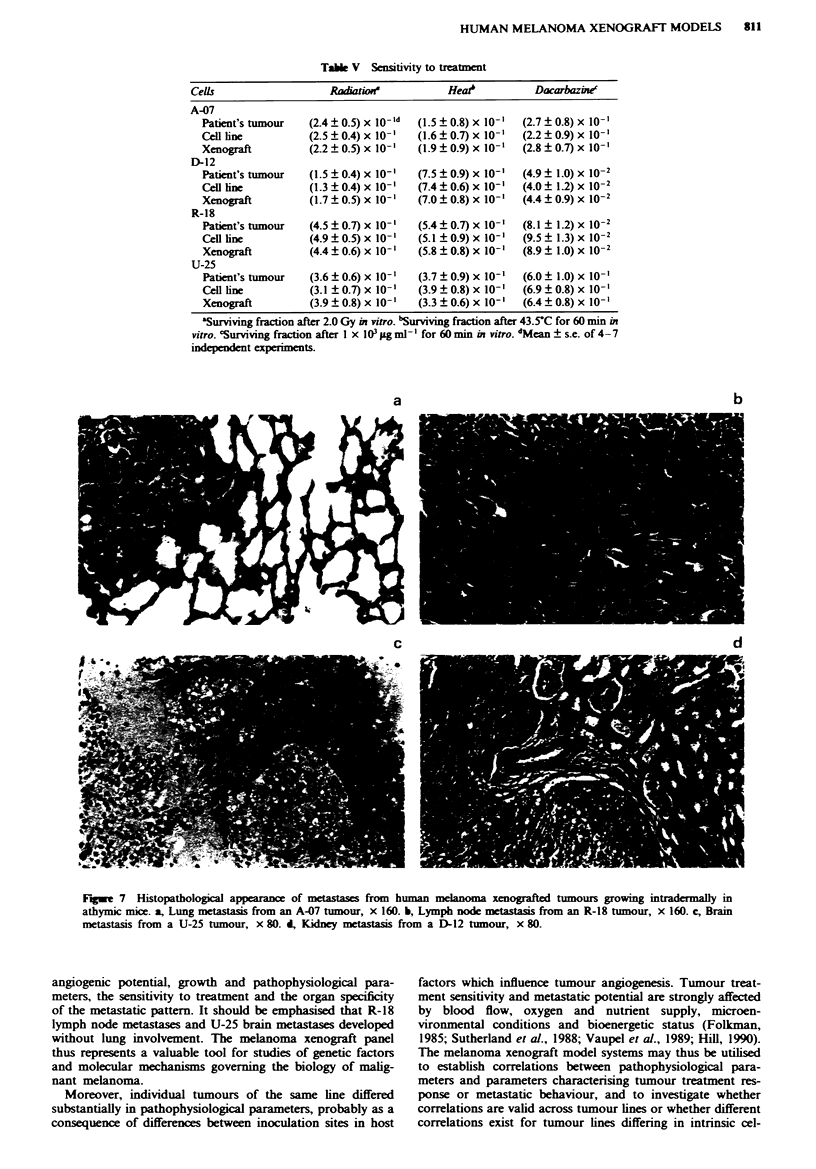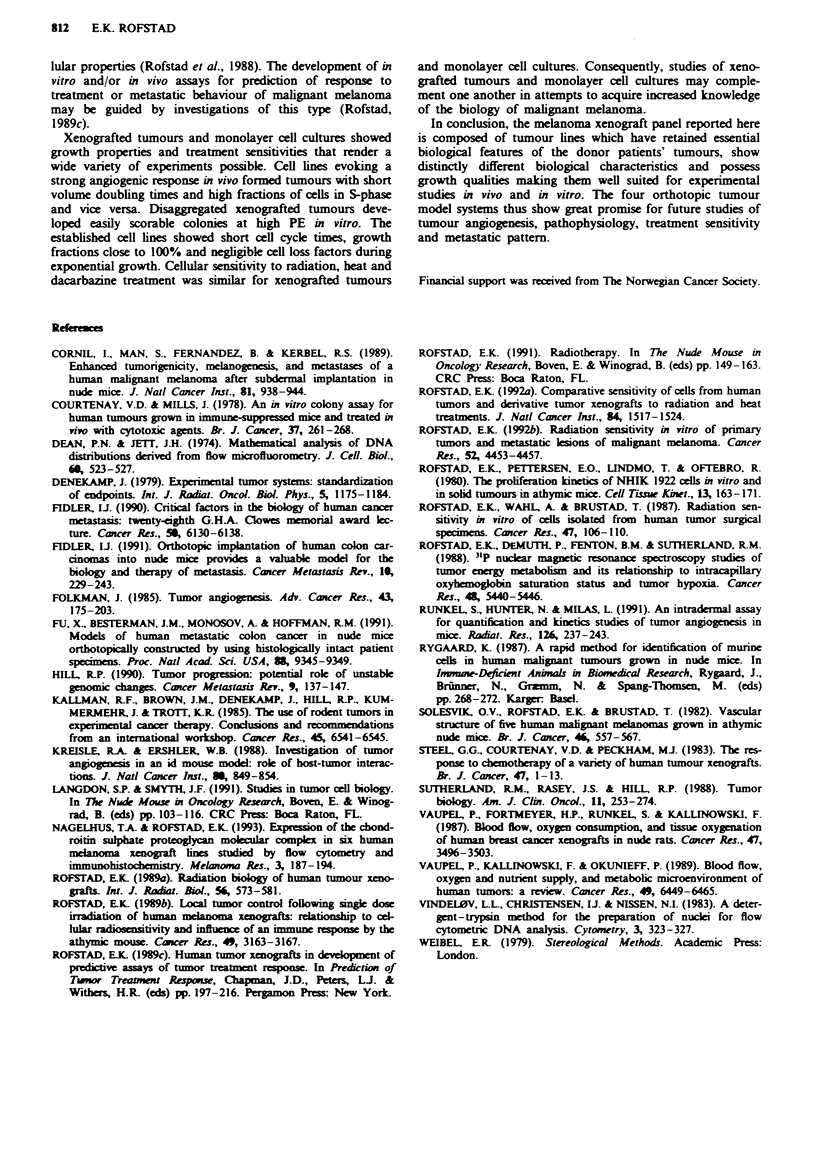# Orthotopic human melanoma xenograft model systems for studies of tumour angiogenesis, pathophysiology, treatment sensitivity and metastatic pattern.

**DOI:** 10.1038/bjc.1994.403

**Published:** 1994-11

**Authors:** E. K. Rofstad

**Affiliations:** Department of Biophysics, Norwegian Radium Hospital, Montebello, Oslo.

## Abstract

**Images:**


Br. J. Cancer (1994), 70, 804-812 © Macmillan Press Ltd., 1994

### Orthotopic human melanoma xenograft model systems for studies of tumour angiogenesis, pathophysiology, treatment sensitivity and metastatic pattern

E.K. Rofstad

*Department of Biophysics, Institute for Cancer Research, The Norwegian Radium Hospital, Montebello, 0310 Oslo, Norway.*

Summary Adequate tumour models are a prerequisite in experimental cancer research. The purpose of the present work was to establish and assess the validity of four new orthotopic human melanoma xenograft model systems (A-07, D-12, R-18, U-25). Permanent cell lines were established in monolayer culture from subcutaneous metastases of four different melanoma patients by using an *in vivo–in vitro* procedure, and cells from these lines were inoculated intradermally in Balb/c *nu/nu* mice to form tumours. Individual xenografted tumours of the same line differed substantially in growth and pathophysiological parameters, probably as a consequence of differences between inoculation sites in host factors which influence tumour angiogenesis. Nevertheless, xenografted tumours of different lines showed distinctly different biological characteristics. Several biological characteristics of the donor patients' tumours were retained in the xenografted tumours, including angiogenic potential; growth, histopathological and pathophysiological parameters; and sensitivity to radiation, heat and dacarbazine treatment. Moreover, the organ-specific metastatic pattern of the xenografted tumours reflected the pattern of distant metastases in the donor patients. The organs of preference for distant metastases were lungs (A-07, D-12), lymph nodes (R-18) and brain (U-25). R-18 lymph node metastases and U-25 brain metastases developed in the absence of lung involvement. The four orthotopic human melanoma xenograft model systems show great promise for future studies of tumour angiogenesis, pathophysiology, treatment sensitivity and metastatic pattern.

Advances in cancer research require appropriate experimental models to explore basic mechanisms affecting the clinical behaviour and treatment sensitivity of tumours. Human models are usually preferable to rodent models; experimental rodent tumours are at best general models of human cancer (Denekamp, 1979), whereas experimental human tumours are supposed to model adequately the specific neoplastic disease of the donor patient (Sutherland *et al.*, 1988). The availability of congenitally athymic mice and severe combined immune deficiency mice has resulted in increased use of human tumour xenografts in cancer research the last two decades. Recent studies have indicated that orthotopic heterotransplantation of human tumours may result in metastatic patterns in mice similar to those observed in man (Fidler, 1991; Fu *et al.*, 1991). Moreover, cellular treatment sensitivity is probably retained during serial transplantation of some human tumours in immune-deficient mice (Rofstad, 1992*a*).

No single xenograft model can be expected to be appropriate for all types of questions related to a specific neoplastic disease; a panel of models of the same histopathological type is required (Kallman *et al.*, 1985). A xenograft panel is particularly useful if it is composed of tumour lines that differ substantially in their biological characteristics and at the same time reflect the biology of the donor patients' tumours. Moreover, the xenografted tumours should have disaggregation and growth properties making them suitable for studies *in vitro* as well as *in vivo*.

Four new orthotopic human melanoma xenograft model systems for studies of tumour angiogenesis, pathophysiology, treatment sensitivity and metastatic pattern are reported in the present communication. Tumour treatment sensitivity and metastatic pattern are governed by intrinsic properties of the tumour cells (Sutherland *et al.*, 1988; Hill, 1990), but are strongly modified by angiogenic and pathophysiological parameters of the tumour tissue (Folkman, 1985; Vaupel *et al.*, 1989). The validity of the model systems was assessed by comparing essential biological characteristics of donor patients' tumours and derivative xenografted tumours.

Received 29 April 1994; and in revised form 28 June 1994.

### Materials and methods

### *Donor patients' tumours*

Four Caucasian patients (A-07, D-12, R-18, U-25) admitted to The Norwegian Radium Hospital for the treatment of malignant melanoma were donors of tumour tissue (Table I). The patients had developed regional metastases at the time of initial diagnosis. The R-18 patient presented with extensive metastatic deposits also in distant lymph nodes. The other patients developed distant metastases subsequent to the initial diagnosis, first in lymph nodes and then in lungs (A-07, D-12) and brain (U-25).

Melanoma tissue was obtained from large regional subcutaneous metastases prior to initiation of chemotherapy. The tumour specimens were processed in RPMI-1640 culture medium at 4°C immediately after the surgery. Blood clots and normal tissues were removed. The cleaned melanoma tissue was divided into pieces of approximately 2 $mm^3$ or 0.2 $cm^3$. Xenografted tumours were established by implanting the 2 $mm^3$ pieces subcutaneously in athymic mice. Histological preparations or single-cell suspensions for biological studies were prepared from the 0.2 $cm^3$ pieces.

### *Cell lines*

Permanent cell lines, one from each of the four patients, were established in monolayer culture. Single-cell suspensions were prepared by mechanical disaggregation of xenografted tumours in the first passage and seeded in 25 $cm^2$ tissue culture flasks containing 5 ml of culture medium (RPMI-1640 medium containing 20% fetal calf serum, 250 mg $l^{-1}$ penicillin and 50 mg $l^{-1}$ streptomycin). The suspensions were incubated at 37°C in a humidified atmosphere of 5% carbon dioxide in air. The cells attached to the bottom of the flasks, and proliferation was initiated within 2 days. The cultures were trypsinised (treatment with 0.05% trypsin/0.02% EDTA solution at 37°C for 2 min) and subcultured at confluence. Murine fibroblasts contaminating the cultures were removed by differential trypsinisation. Chromosome analysis revealed that cultures in passage 30 were free from murine stromal cells. The cultures showed slow and irregular growth during the first 35 passages *in vitro*. The rate of cell proliferation was

**Table I** Tumour characteristics in donor patients

| *Tumour* | *Patient age (year)* | *Patient sex* | *Primary tumour* | *Breslow thickness (mm)* | *Regional metastases* | *Distant metastases* |
|---|---|---|---|---|---|---|
| A-07 | 50 | Male | Upper limb | 3.8 | Subcutis<br>Lymph node | Lymph node<br>Lung |
| D-12 | 38 | Female | Trunk | 8.3 | Subcutis<br>Lymph node | Lymph node<br>Lung |
| R-18 | 45 | Female | Neck | 7.4 | Subcutis<br>Lymph node | Subcutis<br>Lymph node |
| U-25 | 49 | Male | Trunk | 5.0 | Subcutis<br>Lymph node | Lymph node<br>Brain |

stable and reproducible beyond passage 50. Large stocks of cells in passage 75 were frozen and stored in liquid nitrogen. These stocks were verified to be free from *Mycoplasma* contamination by using both the Hoechst fluorescence and mycotrin methods.

The cellular parameters reported here were determined by studies of exponentially growing cultures in passages 75–100, i.e. they were determined after stable, permanent cell lines had been established. The cells were in this period cultured in RPMI-1640 medium (25 mM HEPES and L-glutamine) supplemented with 13% fetal calf serum, 250 mg $l^{-1}$ penicillin and 50 mg $l^{-1}$ streptomycin. The cultures were incubated at 37°C in a humidified atmosphere of 5% carbon dioxide in air and subcultured three times a week.

Cell number doubling time was determined by seeding $5 \times 10^4$ cells in 80 $cm^2$ tissue culture flasks and measuring number of cells per flask as a function of time. Growth fraction and cell loss factor were determined by observing 40 randomly selected cells in a 48 h period; fraction of cells entering mitosis and the outcome of mitosis were registered (Rofstad *et al.*, 1980). Plating efficiency (PE) was measured by seeding 100 cells in 25 $cm^2$ tissue culture flasks containing approximately $1.0 \times 10^5$ heavily irradiated (30 Gy) feeder cells. DNA index was determined by flow cytometry (see below).

*Xenografted tumours*

Adult Balb/c *nu/nu* mice, bred at our research institute, were used as host animals for xenografted tumours. The mice were maintained under specific pathogen-free conditions at constant temperature (24–26°C) and humidity (30–50%). Sterilised food and tap water were given *ad libitum*.

Approximately $3.5 \times 10^5$ cells in 10 µl of $Ca^{2+}$- and $Mg^{2+}$-free Hanks' balanced salt solution (HBSS) were inoculated intradermally in the flanks of the mice by using a 100 µl Hamilton syringe. The cells were harvested from exponentially growing cultures in passages 75–100 by trypsinisation. The take rate was 100%. Tumour volume ($V$) was calculated as $V = \pi/6 \times ab^2$, where $a$ is the longer and $b$ the shorter of two perpendicular tumour diameters, measured with callipers. Volume doubling time, fraction of necrotic tissue, fraction of cells in S-phase and treatment sensitivity were determined for tumours with volumes within the range of 200–400 $mm^3$. Tumour weight was determined immediately after tumour excision and refers to the wet weight.

*Histopathological examinations*

Tumour samples were fixed in phosphate-buffered 4% paraformaldehyde, dehydrated, embedded in paraffin casts, cut in 6-µm-thick sections and stained. Haematoxylin and eosin staining were used for ordinary histopathological examinations and for measurement of fraction of necrotic tissue. Standard immunoperoxidase procedures were used to highlight vessels in donor patients' tumours and to visualise extracellular matrix in xenografted tumours (Nagelhus & Rofstad, 1993). Polyclonal rabbit anti-factor VIII (Dakopatts), polyclonal rabbit anti-laminin (Serotec), polyclonal rabbit anti-fibronectin (Calbiochem) and monoclonal mouse anti-type IV collagen (Dakopatts) were used as primary reagents. The sections were stained by using diaminobenzidine as substrate and haematoxylin for counterstaining. Murine host cells in xenografted tumours were identified by using the bisbenzimide staining technique (Rygaard, 1987).

Vascular density was assessed at a magnification of × 200 by counting number of capillary profiles per field of view, corresponding to 0.75 $mm^2$. A structure was defined as a countable capillary only if a lumen and at least one brown-staining endothelial cell were identified. Volume fraction of necrosis was determined by point counting (Solesvik *et al.*, 1982). Five fields of view, selected according to stringent stereological criteria (Weibel, 1979), were analysed in each of 4–6 different tumour pieces from each patient.

*Ultrastructural examinations*

The ultrastructure of microvessels in xenografted tumours was examined by transmission electron microscopy. Tumour pieces were fixed in a cacodylate-buffered mixture of 1% glutaraldehyde and 4% paraformaldehyde, post-fixed in buffered 1% osmium tetroxide and dehydrated in graded alcohol solutions. The tissue was embedded in Epon–Araldite and polymerised at 80°C. Ultrathin sections were double stained with uranyl acetate and lead citrate.

*Cell suspensions*

Single-cell suspensions were prepared from donor patients' tumours and xenografted tumours by using a standardised mechanical and enzymatic procedure. The tumour tissue was minced with crossed scalpels in cold HBSS prior to enzymatic treatment at 37°C for 2 h. The enzyme solution consisted of 0.2% collagenase, 0.05% pronase and 0.02% DNAse in HBSS. The fraction of contaminating diploid host cells in the suspensions, determined by flow cytometry, was low for both donor patients' tumours (<10%) and xenografted tumours (<1%). The fraction of morphologically intact tumour cells, determined by trypan blue exclusion, was always high (>80%). Cell aggregates were removed from the suspensions by filtration through 30 µm nylon mesh.

*Flow cytometry*

An Argus Skatron flow cytometer, equipped with an arch lamp as excitation source, was used for measurements of DNA histograms. Suspensions of clean cell nuclei were prepared from single-cell suspensions and stained with propidium iodide according to the detergent–trypsin method (Vindeløv *et al.*, 1983). Excitation of propidium iodide was accomplished by using the 546 nm mercury line. Fluorescence was detected at wavelengths above 590 nm. Chicken and trout red blood cells were used as internal reference standards. DNA index was calculated by using human lymphocytes as diploid reference. The DNA histograms were analysed mathematically to determine the distribution of cells in the cell cycle (Dean & Jett, 1974).

*Treatment sensitivity*

Single cells, kept in suspension in RPMI-1640 culture medium at pH 7.4, were treated with radiation (2.0 Gy), heat (43.5°C for 60 min) or dacarbazine ($1 \times 10^3 \mu g\ ml^{-1}$ for 60 min). Radiation treatment was carried out under aerobic conditions at room temperature at a dose rate of 3.4 Gy $min^{-1}$. The X-ray unit was operated at 220 kV, 20 mA and with 0.5 mm copper filtration (Rofstad, 1992*b*). Heat treatment was performed by using a thermostatically regulated water bath. To avoid enhancement of the cell inactivation by the enzymes used for the preparation of single-cell suspensions, the cells were incubated at 37°C in an atmosphere of 5% carbon dioxide in air for 3 h prior to the heat exposure (Rofstad, 1992*a*). Dacarbazine treatment was given by incubating the cells at 37°C in an atmosphere of 5% carbon dioxide in air in the presence of the drug. The cells were washed in $Ca^{2+}$- and $Mg^{2+}$-free HBSS after treatment.

Cell survival was measured by using a soft-agar colony assay (Courtenay & Mills, 1978). The two distinguishing features of the assay are (a) the use of growth factors from rat erythrocytes to enhance the PE and (b) the use of a tissue-mimicking atmosphere of 3% oxygen, 5% carbon dioxide and 92% nitrogen to increase the rate of cell proliferation. Details of the procedure are reported elsewhere (Rofstad *et al.*, 1987). Macrophages are the only host cells that form colonies in the assay. The loosely packed macrophage colonies were easily distinguished from the densely packed melanoma colonies. Melanoma colonies containing more than 50 cells were counted by using a stereomicroscope (Rofstad *et al.*, 1987). The PE of trypan blue excluding melanoma cells was 10–25% (donor patients' tumours), 10–40% (xenografted tumours) and 80–95% (monolayer cultures).

*Angiogenesis*

Tumour angiogenesis was assessed by using the intradermal angiogenesis assay (Kreisle & Ershler, 1988; Runkel *et al.*, 1991). Approximately $3.5 \times 10^5$ cells, harvested from cultures in exponential growth, were inoculated intradermally in the flanks of mice as described above. The skin around the inoculation sites was removed at predetermined times after the inoculation. The tumours were located with a dissecting microscope, and angiogenesis was quantified by counting the number of capillaries oriented toward the tumours. The number of capillaries was corrected for the background, determined after injection of 10 µl of HBSS. The tumours were dissected free from the skin and weighed after the capillaries were scored.

*Metastatic pattern*

Intradermal tumours, 30 of each of the four lines, were initiated in 120 mice as described above. The tumours were removed surgically as soon as the largest tumour diameter attained 10 mm, and the wounds were closed with surgical clips. The mice were killed and autopsied 6 months after the primary tumour was removed or when they were moribund. All organs were examined for macroscopic metastases. Lungs, axial and inguinal lymph nodes, brain, kidneys, adrenal glands, pancreas and liver were subjected to histological assessment of metastases. Ten sections from different locations in each organ, prepared and stained with haemotoxylin and eosin, as described above, were examined.

*Statistical analysis*

Statistical comparisons of data were performed by non-parametric analysis using the Mann–Whitney *U*-test and the Kruskal–Wallis *H*-test. A significance criterion of $P < 0.05$ was used.

### Results

*Metastatic pattern in donor patients*

The location of the primary tumour and the metastatic pattern differed between the four donor patients (Table I). All patients developed regional metastases in subcutis and lymph nodes and distant metastases in lymph nodes, irrespective of the location of the primary tumour. The distant lymph node metastases of the R-18 patient were particularly aggressive; they occurred within the thorax and the abdomen and invaded adjacent tissues. The patients died 16–48 weeks after presentation. The causes of death resulted from distant metastases involving lungs (A-07, D-12), lymph nodes (R-18) and brain (U-25).

*Histopathology, growth and pathophysiology of donor patients' tumours*

The donor patients' tumours were heterogeneous in general histopathological appearance. The tissue showed a solid growth pattern separated by a meshwork of fibrous septae. The density of the meshwork and the thickness of the septae differed substantially between tumour regions. Extensive spatial heterogeneity with respect to cell type, cellular pleomorphism, nuclear atypia, nucleolar prominence and mitotic activity was also a characteristic feature of the tissue. Significant differences between the four tumours were not detected. They were all judged to be highly malignant by histopathological criteria.

The donor patients' tumours were also heterogeneous in growth and pathophysiological parameters (Table II). Fraction of necrotic tissue, fraction of cells in S-phase and vascular density differed between tumour regions and between tumours. Tumours showing high vascular densities also showed high fractions of cells in S-phase and vice versa. The tumour sequence from high to low values of these parameters was: A-07, D-12, R-18, U-25 [fraction of cells in S-phase: A-07 *vs* D-12, D-12 *vs* R-18 ($P < 0.01$), R-18 *vs* U-25 ($P < 0.05$); vascular density: A-07 *vs* D-12, D-12 *vs* R-18 ($P < 0.05$), R-18 *vs* U-25 (NS, $P \sim 0.1$)]. There were no correlations between vascular density or fraction of cells in S-phase on the one hand and fraction of necrotic tissue on the other. The fraction of necrotic tissue was significant in most regions of the D-12 and U-25 tumours, but small or zero in the A-07 and R-18 tumours.

*Growth of cell lines*

The four cell lines were characterised by extensive cellular pleomorphism. Most cells were polygonal or spindle shaped and showed abundant cytoplasm and large irregular nuclei with prominent nucleoli. DNA index differed significantly between the cell lines, whereas most growth parameters were similar (Table III). The cell lines were hyperdiploid; DNA index ranged from 1.1 ± 0.1 to 1.8 ± 0.1. Growth fraction was high (>90%) and cell loss factor was low (<10%) during exponential growth. The cell lines formed easily scorable colonies within 7–10 days after plating and showed a high PE (>80%). Median cell cycle time during exponential growth, calculated from cell number doubling time, growth fraction and cell loss factor, was slightly longer for

**Table II** Growth and pathophysiological parameters of tumours in donor patients

| Tumour | *Necrotic fraction (%)* | *S-phase fraction (%)* | *Vascular density* |
|---|---|---|---|
| A-07 | 0–5[a] | 18–26[b] | 82–280[c] |
| D-12 | 6–22 | 11–16 | 35–196 |
| R-18 | 0–12 | 6–12 | 17–158 |
| U-25 | 15–43 | 4–9 | 21–133 |

[a]Range of 4–6 different tumour pieces. [b]Range of 4–5 different tumour pieces. [c]Number of capillary profiles per field of view. Range of six different tumour pieces.

the R-18 and U-25 lines than for the A-07 and D-12 lines ($P<0.05$).

### *Angiogenesis of xenografted tumours*

Inoculation of tumour cells evoked a strong angiogenic response in the mice (Figure 1). Tumour weight and number of capillaries oriented towards the tumours increased with time after inoculation (Figure 2). The rate of angiogenesis and the increase in tumour weight with time were tumour line dependent and paralleled one another. The sequence of the tumour lines from high to low values of these two parameters was: A-07, D-12, R-18, U-25 [tumour weight: A-07 *vs* D-12, D-12 *vs* R-18, R-18 *vs* U-25 ($P<0.005$); number of capillaries: A-07 *vs* D-12, D-12 *vs* R-18 ($P<0.01$), R-18 *vs* U-25 ($P<0.05$)].

The angiogenic response was heterogeneous also for cells of the same line (Figure 3). This heterogeneity was reflected in the rate of angiogenesis and in the density of the neovasculature. The number of capillaries oriented towards the tumours at day 7 after inoculation was used as a parameter for the rate of angiogenesis (Figure 3a). The number of capillaries oriented toward the tumours at tumour weights of approximately 30 mg was used as a parameter for the density of the neovasculature (Figure 3b). The latter parameter might also reflect the capillary density within the 30 mg tumours.

**Table III** Growth parameters of cell lines

| *Line* | *DNA index* | *GF (%)*[a] | *φ (%)*[b] | *PE (%)*[c] | $T_c$ *(h)*[d] |
|---|---|---|---|---|---|
| A-07 | 1.8 ± 0.1[e] | 90–100[f] | 0–10[f] | 80–100[f] | 15–17[f] |
| D-12 | 1.1 ± 0.1 | 95–100 | 0–5 | 90–100 | 15–17 |
| R-18 | 1.4 ± 0.1 | 95–100 | 0–5 | 90–100 | 19–21 |
| U-25 | 1.6 ± 0.1 | 90–100 | 0–10 | 80–100 | 19–21 |

[a]GF, growth fraction. [b]φ, cell loss factor. [c]PE, plating efficiency. [d]$T_c$, cell cycle time. [e]Mean ± s.e. of five independent experiments. [f]Range of five independent experiments.

**Figure 1** Angiogenic response in athymic mice following intradermal inoculation of $3.5\times10^5$ human melanoma cells. **a**, A-07 tumour 7 days after inoculation. **b**, U-25 tumour 28 days after inoculation.

### *Histopathology, growth and pathophysiology of xenografted tumours*

The xenografted tumours were deposited above the subcutaneous muscle tissue in the deeper part of the dermis, and were not circumscribed by a fibrous capsule (Figure 4a). The dermis (Figure 4b) and the subcutaneous muscle (Figure 4c) were infiltrated and gradually replaced by tumour tissue dur-

**Figure 2** Tumour growth and angiogenesis in athymic mice following intradermal inoculation of $3.5\times10^5$ human melanoma cells. **a**, Tumour wet weight versus time after inoculation. **b**, Number of capillaries oriented toward the tumours versus time after inoculation. Points, mean values. Bars, s.e. of 12–16 tumour inoculations.

ing tumour growth. Finally, the tumours penetrated the epidermis and formed an ulcer. The histopathological appearance of the tumour parenchyma was similar to that of the donor patients' tumours. The extent of cellular pleomorphism and nuclear atypia differed between tumour regions and individual tumours. Distinct differences between the four lines were not detected. Large tumours frequently developed regions with corded growth. The cords, i.e. cylindrical structures of viable tumour cells surrounding functional vessels, were sometimes separated completely by necrosis (Figure 4d).

The xenografted tumours developed a heterogeneous stroma that showed clear histopathological similarities to the stroma of the donor patients' tumours. The vascular network consisted of two distinct types of microvessels: (a) normal microvessels recruited from the pre-existing network in the skin during tumour growth and (b) pathological microvessels

**Figure 3** Angiogenesis in athymic mice following intradermal inoculation of 3.5 x $10^5$ human melanoma cells. **a**, Number of capillaries oriented toward the tumours at day 7 after inoculation. **b**, Number of capillaries oriented toward the tumours at tumour wet weights of approximately 30 mg. Points, single tumour inoculations.

recruited by tumour-induced neovascularisation. The normal microvessels included terminal arterioles invested in vascular smooth muscle, non-fenestrated capillaries and dilated post-capillary venules. The neovasculature showed severe structural abnormalities including incomplete endothelial lining, interrupted or absent basement membrane and/or lack of pericytes. The extracellular matrix appeared in immunohistochemical preparations as an irregular meshwork, differing considerably in structure and density between tumour regions and individual tumours (Figure 5). Antibodies against laminin, fibronectin and type IV collagen gave similar staining patterns. The infiltration of host cells in the tumours was sparse; leucocytes, macrophages and fibroblasts constituted less than 1% of the total number of cells. Significant differences between the four lines in the appearance of the stroma were not detected.

Quantitative analysis showed that tumour lines and individual tumours of the same line differed substantially in growth and pathophysiological parameters (Figure 6). The differences were reflected in tumour volume doubling time, fraction of necrotic tissue and fraction of cells in S-phase. The sequence of the tumour lines from short to long volume doubling times was equal to that from high to low fractions of cells in S-phase: A-07, D-12, R-18, U-25 [volume doubling time: D-12 *vs* R-18 ($P<0.01$), A-07 *vs* D-12, R-18 *vs* U-25 ($P<0.05$); fraction of cells in S-phase: A-07 *vs* D-12, D-12 *vs* R-18 ($P<0.05$), R-18 *vs* U-25 (NS, $P\sim0.2$)]. There were no correlations between volume doubling time or fraction of cells in S-phase on the one hand and fraction of necrotic tissue on the other. Fraction of necrotic tissue was significant in most D-12 and U-25 tumours, but small or zero in A-07 and R-18 tumours.

### *Metastatic pattern of xenografted tumours*

The xenografted tumours showed organ-specific metastatic patterns (Table IV). The organs of preference for the development of metastases were lungs for A-07 and D-12 tumours (Figure 7a), lymph nodes for R-18 tumours (Figure 7b) and brain for U-25 tumours (Figure 7c). A-07 tumours grew faster and were more angiogenic than D-12 tumours, but did not metastasise more frequently than D-12 tumours. U-25 tumours grew more slowly and were less angiogenic than A-07, D-12 and R-18 tumours, but were the only ones that formed brain metastases. R-18 lymph node metastases and U-25 brain metastases developed without the occurrence of lung metastases. Approximately 15% of the mice developed organ-specific metastases within 6 months after the inoculation of the primary tumour. In addition, approximately 10% of the mice developed abdominal metastases. Different organs in the abdomen were involved, particularly adrenal glands, pancreas, liver and kidneys (Figure 7d). In contrast, xenografted tumours grown at subcutaneous sites did not develop organ-specific or abdominal metastases.

### *Treatment sensitivity of donor patients' tumours, cell lines and xenografted tumours*

The donor patient's tumour, the corresponding cell line and the corresponding xenografted tumours showed similar sensitivities to treatment *in vitro*, no matter which line or treatment modality was considered (Table V). The sensitivity to a given treatment differed substantially between the lines. There were no correlations between the sensitivity to radiation treatment, heat treatment and dacarbazine treatment. The sequences of the lines from high to low cell surviving fractions following treatment were: R-18, U-25, A-07, D-12 (radiation treatment); D-12, R-18, U-25, A-07 (heat treatment); and U-25, A-07, R-18, D-12 (dacarbazine treatment).

### Discussion

Human tumour xenografts are supposed to be appropriate models for addressing specific questions related to the clinical

**Figure 4** Histopathological appearance of human melanoma xenografted tumours growing intradermally in athymic mice. **a**, Tumour localisation and appearance shortly after inoculation, D-12 tumour, × 40. **b**, Tumour infiltration in the dermis, D-12 tumour, × 160. **c**, Tumour infiltration in the subcutaneous muscle, D-12 tumour, × 160. **d**, Tumour cord surrounded by necrosis, D-12 tumour, × 80.

behaviour and treatment sensitivity of human tumours. Several studies have suggested that fundamental biological properties of the donor patients' tumours may be retained after heterotransplantation, including expression of proto-oncogenes, tumour-associated antigens and receptors for growth factors and hormones (Langdon & Smyth, 1991); vascular and microenvironmental parameters (Vaupel *et al.*, 1987); sensitivity to drug, radiation and heat treatment (Steel *et al.*, 1983; Rofstad, 1989*a*); and growth pattern and metastatic potential (Fidler, 1990). However, biological conditions which may limit the usefulness of human tumour xenografts in cancer research have also been recognised. Thus, the vascular network and the blood of xenografted tumours originate from the host, a fact which may cause the oxygen and nutrient supply to differ from that in tumours in man (Solesvik *et al.*, 1982). Moreover, immune reactions by the host may be active against xenografted tumours and artificially enhance the response to treatment (Rofstad, 1989*b*).

Research groups should endeavour to have available a panel of xenograft lines of the same histopathological type (Kallman *et al.*, 1985; Sutherland *et al.*, 1988; Rofstad, 1991). The panel should be composed of lines which (a) have retained essential biological features of the donor patients' tumours, (b) show distinctly different biological characteristics in the new host and (c) possess growth qualities making them well suited for experimental studies *in vivo* and *in vitro*. These recommendations are met by the melanoma xenograft panel reported here.

Retention of biological characteristics in heterotransplanted human tumours may require the interaction of the tumour cells with the relevant organ microenvironment (Fidler, 1990). Orthotopic inoculation seems to be particularly important for accurate reproduction of the metastatic behaviour of human tumours (Fidler, 1991). Thus, intradermal inoculation of human melanoma cells in athymic mice resulted in primary tumours that metastasised at high frequency to draining lymph nodes, whereas the primary tumours that developed after subcutaneous inoculation of the same cells rarely gave rise to lymph node metastases (Cornil *et al.*, 1989). Melanocytes are normally found in the dermal–epidermal junction of the skin. Tumour growth in orthotopic sites was achieved in the present work by intradermal inoculation of melanoma cells; intradermal inoculation resulted in tumours that infiltrated the epidermis of the mice within a short time.

Most reports dealing with retention of biological characteristics in heterotransplanted human tumours refer to studies of a single biological feature or a few closely related biological phenomena. The biological properties of the xenografted tumours were usually compared with the general clinical behaviour of tumours of the same histopathological type. However, critical examination requires measurement of several unrelated biological parameters in donor patients' tumours and derivative xenografted tumours, followed by paired analysis of the data. This approach was used in the present work.

Essential biological features of the donor patients' tumours

**Figure 5** Immunohistochemical visualisation of the extracellular matrix of human melanoma xenografted tumours growing intradermally in athymic mice. **a**, Irregular fine-meshed network, laminin staining of an R-18 tumour, × 80. **b**, Irregular coarse-meshed network, laminin staining of an R-18 tumour, × 80.

**Table IV** Metastatic pattern of xenografted tumours

| *Tumour* | *Abdomen* | *Lungs* | *Lymph nodes* | *Brain* |
|---|---|---|---|---|
| A-07 | 3/30[a] | 4/30 | 0/30 | 0/30 |
| D-12 | 2/30 | 6/30 | 0/30 | 0/30 |
| R-18 | 4/30 | 0/30 | 5/30 | 0/30 |
| U-25 | 2/30 | 0/30 | 0/30 | 5/30 |

[a]Fraction of mice with metastases.

were found to be retained after the heterotransplantation. Thus, xenografted tumours and donor patients' tumours were similar in respect of general histopathological appearance, fraction of necrotic tissue, fraction of cells in S-phase and cellular sensitivity to radiation, heat and dacarbazine treatment. The tumour sequence from high to low values of vascular and angiogenic parameters was equal in mice and patients. Moreover, the organ-specific metastatic pattern of the xenografted tumours reflected the pattern of distant metastases in the donor patients, i.e. each individual cell line generated xenografted tumours that recapitulated the metastatic pattern of the donor tumour tissue. Consequently, basic biological mechanisms relevant for the clinical behaviour and treatment sensitivity of malignant melanoma may be revealed by the use of these melanoma xenograft model systems in cancer research.

The four tumour lines showed distinctly different biological characteristics, i.e. the intrinsic properties of the tumour cells differed between the lines. This was reflected in the

**Figure 6** Growth and pathophysiological parameters of human melanoma xenografted tumours growing intradermally in athymic mice. **a**, Tumour volume doubling time. **b**, Fraction of necrotic tissue. **c**, Fraction of cells in S-phase. Points, single tumours.

**Table V** Sensitivity to treatment

| *Cells* | *Radiation*[a] | *Heat*[b] | *Dacarbazine*[c] |
|---|---|---|---|
| A-07 | | | |
| Patient's tumour | $(2.4 \pm 0.5) \times 10^{-1}$[d] | $(1.5 \pm 0.8) \times 10^{-1}$ | $(2.7 \pm 0.8) \times 10^{-1}$ |
| Cell line | $(2.5 \pm 0.4) \times 10^{-1}$ | $(1.6 \pm 0.7) \times 10^{-1}$ | $(2.2 \pm 0.9) \times 10^{-1}$ |
| Xenograft | $(2.2 \pm 0.5) \times 10^{-1}$ | $(1.9 \pm 0.9) \times 10^{-1}$ | $(2.8 \pm 0.7) \times 10^{-1}$ |
| D-12 | | | |
| Patient's tumour | $(1.5 \pm 0.4) \times 10^{-1}$ | $(7.5 \pm 0.9) \times 10^{-1}$ | $(4.9 \pm 1.0) \times 10^{-2}$ |
| Cell line | $(1.3 \pm 0.4) \times 10^{-1}$ | $(7.4 \pm 0.6) \times 10^{-1}$ | $(4.0 \pm 1.2) \times 10^{-2}$ |
| Xenograft | $(1.7 \pm 0.5) \times 10^{-1}$ | $(7.0 \pm 0.8) \times 10^{-1}$ | $(4.4 \pm 0.9) \times 10^{-2}$ |
| R-18 | | | |
| Patient's tumour | $(4.5 \pm 0.7) \times 10^{-1}$ | $(5.4 \pm 0.7) \times 10^{-1}$ | $(8.1 \pm 1.2) \times 10^{-2}$ |
| Cell line | $(4.9 \pm 0.5) \times 10^{-1}$ | $(5.1 \pm 0.9) \times 10^{-1}$ | $(9.5 \pm 1.3) \times 10^{-2}$ |
| Xenograft | $(4.4 \pm 0.6) \times 10^{-1}$ | $(5.8 \pm 0.8) \times 10^{-1}$ | $(8.9 \pm 1.0) \times 10^{-2}$ |
| U-25 | | | |
| Patient's tumour | $(3.6 \pm 0.6) \times 10^{-1}$ | $(3.7 \pm 0.9) \times 10^{-1}$ | $(6.0 \pm 1.0) \times 10^{-1}$ |
| Cell line | $(3.1 \pm 0.7) \times 10^{-1}$ | $(3.9 \pm 0.8) \times 10^{-1}$ | $(6.9 \pm 0.8) \times 10^{-1}$ |
| Xenograft | $(3.9 \pm 0.8) \times 10^{-1}$ | $(3.3 \pm 0.6) \times 10^{-1}$ | $(6.4 \pm 0.8) \times 10^{-1}$ |

[a]Surviving fraction after 2.0 Gy *in vitro*. [b]Surviving fraction after 43.5°C for 60 min *in vitro*. [c]Surviving fraction after $1 \times 10^3$ μg $ml^{-1}$ for 60 min *in vitro*. [d]Mean ± s.e. of 4–7 independent experiments.

**Figure 7** Histopathological appearance of metastases from human melanoma xenografted tumours growing intradermally in athymic mice. **a**, Lung metastasis from an A-07 tumour, × 160. **b**, Lymph node metastasis from an R-18 tumour, × 160. **c**, Brain metastasis from a U-25 tumour, × 80. **d**, Kidney metastasis from a D-12 tumour, × 80.

angiogenic potential, growth and pathophysiological parameters, the sensitivity to treatment and the organ specificity of the metastatic pattern. It should be emphasised that R-18 lymph node metastases and U-25 brain metastases developed without lung involvement. The melanoma xenograft panel thus represents a valuable tool for studies of genetic factors and molecular mechanisms governing the biology of malignant melanoma.

Moreover, individual tumours of the same line differed substantially in pathophysiological parameters, probably as a consequence of differences between inoculation sites in host factors which influence tumour angiogenesis. Tumour treatment sensitivity and metastatic potential are strongly affected by blood flow, oxygen and nutrient supply, microenvironmental conditions and bioenergetic status (Folkman, 1985; Sutherland *et al.*, 1988; Vaupel *et al.*, 1989; Hill, 1990). The melanoma xenograft model systems may thus be utilised to establish correlations between pathophysiological parameters and parameters characterising tumour treatment response or metastatic behaviour, and to investigate whether correlations are valid across tumour lines or whether different correlations exist for tumour lines differing in intrinsic cellular properties (Rofstad *et al.*, 1988). The development of *in vitro* and/or *in vivo* assays for prediction of response to treatment or metastatic behaviour of malignant melanoma may be guided by investigations of this type (Rofstad, 1989*c*).

Xenografted tumours and monolayer cell cultures showed growth properties and treatment sensitivities that render a wide variety of experiments possible. Cell lines evoking a strong angiogenic response *in vivo* formed tumours with short volume doubling times and high fractions of cells in S-phase and vice versa. Disaggregated xenografted tumours developed easily scorable colonies at high PE *in vitro*. The established cell lines showed short cell cycle times, growth fractions close to 100% and negligible cell loss factors during exponential growth. Cellular sensitivity to radiation, heat and dacarbazine treatment was similar for xenografted tumours and monolayer cell cultures. Consequently, studies of xenografted tumours and monolayer cell cultures may complement one another in attempts to acquire increased knowledge of the biology of malignant melanoma.

In conclusion, the melanoma xenograft panel reported here is composed of tumour lines which have retained essential biological features of the donor patients' tumours, show distinctly different biological characteristics and possess growth qualities making them well suited for experimental studies *in vivo* and *in vitro*. The four orthotopic tumour model systems thus show great promise for future studies of tumour angiogenesis, pathophysiology, treatment sensitivity and metastatic pattern.

Financial support was received from The Norwegian Cancer Society.

### References

CORNIL, I., MAN, S., FERNANDEZ, B. & KERBEL, R.S. (1989). Enhanced tumorigenicity, melanogenesis, and metastases of a human malignant melanoma after subdermal implantation in nude mice. *J. Natl Cancer Inst.*, **81**, 938–944.

COURTENAY, V.D. & MILLS, J. (1978). An *in vitro* colony assay for human tumours grown in immune-suppressed mice and treated *in vivo* with cytotoxic agents. *Br. J. Cancer*, **37**, 261–268.

DEAN, P.N. & JETT, J.H. (1974). Mathematical analysis of DNA distributions derived from flow microfluorometry. *J. Cell. Biol.*, **60**, 523–527.

DENEKAMP, J. (1979). Experimental tumor systems: standardization of endpoints. *Int. J. Radiat. Oncol. Biol. Phys.*, **5**, 1175–1184.

FIDLER, I.J. (1990). Critical factors in the biology of human cancer metastasis: twenty-eighth G.H.A. Clowes memorial award lecture. *Cancer Res.*, **50**, 6130–6138.

FIDLER, I.J. (1991). Orthotopic implantation of human colon carcinomas into nude mice provides a valuable model for the biology and therapy of metastasis. *Cancer Metastasis Rev.*, **10**, 229–243.

FOLKMAN, J. (1985). Tumor angiogenesis. *Adv. Cancer Res.*, **43**, 175–203.

FU, X., BESTERMAN, J.M., MONOSOV, A. & HOFFMAN, R.M. (1991). Models of human metastatic colon cancer in nude mice orthotopically constructed by using histologically intact patient specimens. *Proc. Natl Acad. Sci. USA*, **88**, 9345–9349.

HILL, R.P. (1990). Tumor progression: potential role of unstable genomic changes. *Cancer Metastasis Rev.*, **9**, 137–147.

KALLMAN, R.F., BROWN, J.M., DENEKAMP, J., HILL, R.P., KUMMERMEHR, J. & TROTT, K.R. (1985). The use of rodent tumors in experimental cancer therapy. Conclusions and recommendations from an international workshop. *Cancer Res.*, **45**, 6541–6545.

KREISLE, R.A. & ERSHLER, W.B. (1988). Investigation of tumor angiogenesis in an id mouse model: role of host-tumor interactions. *J. Natl Cancer Inst.*, **80**, 849–854.

LANGDON, S.P. & SMYTH, J.F. (1991). Studies in tumor cell biology. In *The Nude Mouse in Oncology Research*, Boven, E. & Winograd, B. (eds) pp. 103–116. CRC Press: Boca Raton, FL.

NAGELHUS, T.A. & ROFSTAD, E.K. (1993). Expression of the chondroitin sulphate proteoglycan molecular complex in six human melanoma xenograft lines studied by flow cytometry and immunohistochemistry. *Melanoma Res.*, **3**, 187–194.

ROFSTAD, E.K. (1989*a*). Radiation biology of human tumour xenografts. *Int. J. Radiat. Biol.*, **56**, 573–581.

ROFSTAD, E.K. (1989*b*). Local tumor control following single dose irradiation of human melanoma xenografts: relationship to cellular radiosensitivity and influence of an immune response by the athymic mouse. *Cancer Res.*, **49**, 3163–3167.

ROFSTAD, E.K. (1989*c*). Human tumor xenografts in development of predictive assays of tumor treatment response. In *Prediction of Tumor Treatment Response*, Chapman, J.D., Peters, L.J. & Withers, H.R. (eds) pp. 197–216. Pergamon Press: New York.

ROFSTAD, E.K. (1991). Radiotherapy. In *The Nude Mouse in Oncology Research*, Boven, E. & Winograd, B. (eds) pp. 149–163. CRC Press: Boca Raton, FL.

ROFSTAD, E.K. (1992*a*). Comparative sensitivity of cells from human tumors and derivative tumor xenografts to radiation and heat treatments. *J. Natl Cancer Inst.*, **84**, 1517–1524.

ROFSTAD, E.K. (1992*b*). Radiation sensitivity *in vitro* of primary tumors and metastatic lesions of malignant melanoma. *Cancer Res.*, **52**, 4453–4457.

ROFSTAD, E.K., PETTERSEN, E.O., LINDMO, T. & OFTEBRO, R. (1980). The proliferation kinetics of NHIK 1922 cells *in vitro* and in solid tumours in athymic mice. *Cell Tissue Kinet.*, **13**, 163–171.

ROFSTAD, E.K., WAHL, A. & BRUSTAD, T. (1987). Radiation sensitivity *in vitro* of cells isolated from human tumor surgical specimens. *Cancer Res.*, **47**, 106–110.

ROFSTAD, E.K., DEMUTH, P., FENTON, B.M. & SUTHERLAND, R.M. (1988). $^{31}P$ nuclear magnetic resonance spectroscopy studies of tumor energy metabolism and its relationship to intracapillary oxyhemoglobin saturation status and tumor hypoxia. *Cancer Res.*, **48**, 5440–5446.

RUNKEL, S., HUNTER, N. & MILAS, L. (1991). An intradermal assay for quantification and kinetics studies of tumor angiogenesis in mice. *Radiat. Res.*, **126**, 237–243.

RYGAARD, K. (1987). A rapid method for identification of murine cells in human malignant tumours grown in nude mice. In *Immune-Deficient Animals in Biomedical Research*, Rygaard, J., Brünner, N., Græmm, N. & Spang-Thomsen, M. (eds) pp. 268–272. Karger: Basel.

SOLESVIK, O.V., ROFSTAD, E.K. & BRUSTAD, T. (1982). Vascular structure of five human malignant melanomas grown in athymic nude mice. *Br. J. Cancer*, **46**, 557–567.

STEEL, G.G., COURTENAY, V.D. & PECKHAM, M.J. (1983). The response to chemotherapy of a variety of human tumour xenografts. *Br. J. Cancer*, **47**, 1–13.

SUTHERLAND, R.M., RASEY, J.S. & HILL, R.P. (1988). Tumor biology. *Am. J. Clin. Oncol.*, **11**, 253–274.

VAUPEL, P., FORTMEYER, H.P., RUNKEL, S. & KALLINOWSKI, F. (1987). Blood flow, oxygen consumption, and tissue oxygenation of human breast cancer xenografts in nude rats. *Cancer Res.*, **47**, 3496–3503.

VAUPEL, P., KALLINOWSKI, F. & OKUNIEFF, P. (1989). Blood flow, oxygen and nutrient supply, and metabolic microenvironment of human tumors: a review. *Cancer Res.*, **49**, 6449–6465.

VINDELØV, L.L., CHRISTENSEN, I.J. & NISSEN, N.I. (1983). A detergent–trypsin method for the preparation of nuclei for flow cytometric DNA analysis. *Cytometry*, **3**, 323–327.

WEIBEL, E.R. (1979). *Stereological Methods*. Academic Press: London.